# Newborn Screening for X-Linked Adrenoleukodystrophy (X-ALD): Biochemical, Molecular, and Clinical Characteristics of Other Genetic Conditions

**DOI:** 10.3390/genes15070838

**Published:** 2024-06-26

**Authors:** Carlos F. Mares Beltran, Christina G. Tise, Rebekah Barrick, Annie D. Niehaus, Rebecca Sponberg, Richard Chang, Gregory M. Enns, Jose E. Abdenur

**Affiliations:** 1 Division of Metabolic Disorders, Children’s Hospital of Orange County (CHOC), Orange, CA 92868, USA; 2 Division of Medical Genetics, Albany Medical Center (AMC), Albany, NY 12208, USA; 3 Division of Medical Genetics, Department of Pediatrics, Lucile Packard Children’s Hospital, Stanford University, Stanford, CA 94304, USA

**Keywords:** X-linked adrenoleukodystrophy (X-ALD), newborn screening (NBS), *ABCD1* gene, Zellweger spectrum disorder (ZSD), *PEX* genes

## Abstract

The state of California (CA) added X-linked adrenoleukodystrophy (X-ALD) to newborn screening (NBS) in 2016 via the measurement of C26:0-lysophosphatidylcholine (C26:0-LPC) in a two-tier fashion, followed by sequencing of the *ABCD1* gene. This has resulted in the identification of individuals with genetic conditions beyond X-ALD that can also result in elevated C26:0-LPC by NBS. We describe the biochemical, molecular, and clinical characteristics of nine patients from two metabolic centers in California who screened positive by NBS for elevated C26:0-LPC between 2016 and 2022 and were ultimately diagnosed with a genetic condition other than X-ALD. Seven individuals were diagnosed with Zellweger spectrum disorder (ZSD) due to biallelic variants in *PEX* genes. One male was diagnosed with Klinefelter syndrome and one female was found to have an X chromosome contiguous gene deletion syndrome after the identification of a heterozygous VUS and hemizygous VUS variant in *ABCD1,* respectively. Patients with ZSD had significantly higher first- and second-tier C26:0-LPC levels compared to the two non-ZSD cases. Identification of children with ZSD and atypical patterns of *ABCD1* variants is a secondary benefit of NBS for X-ALD, leading to earlier diagnosis, prompt therapeutic initiation, and more accurate genetic counseling. As screening for X-ALD continues via the measurement of C26:0-LPC, our knowledge of additional genetic conditions associated with elevated C26:0-LPC will continue to advance, allowing for increased recognition of other genetic disorders for which early intervention is warranted.

## 1. Introduction

The main goal of NBS is to identify disorders that are life-threatening and have potential treatment before they become symptomatic to prevent morbidity and death. Inborn metabolic diseases are a large group of disorders that are included in newborn screening programs as many of them meet the above criteria.

In 2015, the Advisory Committee on Heritable Disorders in Newborns and Children added X-linked adrenoleukodystrophy (X-ALD) to the Recommended Uniform Screening Panel (RUSP), the list of primary conditions to be screened by the state newborn screening programs across the United States. The State of California was one of the first in the United States to implement screening for X-ALD in February 2016 via the measurement of C26:0-lysophosphatidylcholine (C26:0-LPC) in a two-tier method, followed by sequencing of the *ABCD1* gene to complete the screening [1]. C26:0-LPC is a biomarker indicative of defective peroxisomal dysfunction, but is not specific for X-ALD [2]. Since 2016, the implementation of screening for X-ALD has resulted in the identification of patients with genetic conditions beyond X-ALD such as Zellweger spectrum disorder (ZSD), Aicardi Goutières syndrome (AGS) [3,4], and even non-genetic conditions such as neonatal lupus [5], which were also detected by increased C26:0-LPC measured by NBS. However, there is still limited information about the characteristics of these patients and further data are needed.

Both X-ALD and Zellweger spectrum disorder (ZSD) are genetic conditions characterized by peroxisomal dysfunction. ZSD is caused by various defects in one of thirteen *PEX* genes, and the group of disorders can have a broad clinical presentation ranging from mild to very severe phenotypes [6]. The *PEX* genes encode proteins involved in the biogenesis and proliferation of peroxisomes [7], and defects in these genes disturb the production and maintenance of normal peroxisomes, affecting multiple metabolic pathways like the catabolism of very-long chain fatty acids (VLCFA), branched-chain fatty acids, plasmalogen synthesis, and bile acid synthesis including cholic acid.

ZSD is suspected in patients presenting with hypotonia, poor feeding, developmental delays, failure to thrive (FTT), hearing loss, and/or visual impairment [8]. Dysmorphic features of affected individuals include frontal bossing and a large anterior fontanelle. Other commonly observed manifestations include hepatomegaly associated with cholestasis, elevation in transaminases, and liver dysfunction. Magnetic resonance imaging (MRI) of the brain can reveal malformations and/or migration defects, especially polymicrogyria. Biochemical features of ZSD include increased plasma levels of very-long chain fatty acids, phytanic and pristanic acids, elevation of pipecolic acid in blood and urine, and decreased blood plasmalogen levels [9]. Given the broad clinical and biochemical spectrum of ZSD, molecular genetic testing is required to confirm a specific diagnosis.

Management of ZSD is primarily supportive and focuses on addressing symptoms and comorbidities associated with the disorder including nutritional support and the monitoring of liver function. Treatment of ZSD may also include addressing the metabolic disturbances associated with impaired bile acid synthesis in the liver by using cholic acid supplementation. Treatment with cholic acid has demonstrated efficacy in ameliorating liver dysfunction, as evidenced by the stabilization of serum liver chemistries [10]. Furthermore, the relatively favorable safety profile underscores the potential of cholic acid as a therapeutic intervention in ZSD. Nonetheless, the effectiveness of bile acid supplementation in ZSD continues to be studied, with current evidence showing a mixed picture regarding its long-term efficacy during follow-up assessments [11].

In this case series, we report the clinical, biochemical, and molecular characteristics of nine patients that were ascertained due to elevations of VLCFA through the State of CA NBS program whose final diagnosis was a genetic condition other than X-ALD.

## 2. Materials and Methods

This study is a collaboration between two metabolic centers in the State of California: Children’s Hospital of Orange County (CHOC) and Lucile Packard Children’s Hospital at Stanford University. Both centers functioned as coordinating investigation sites. IRB approval was obtained at both centers (IRB # 22069). Our cohort comprised a total of nine patients (five from CHOC, four from Stanford) that were identified between February 2016 and December 2022.

The State of California screens for X-ALD by measuring VLCFA, specifically C26:0-lysophosphatidylcholine (C26:0-LPC), in a two-tier fashion. The first tier utilizes flow injection tandem mass spectrometry (FIA-MS/MS) to measure C26:0-LPC. The second tier employs liquid chromatography tandem mass spectrometry (LC-MS/MS) to yield a more accurate level of C26:0-LPC. If the first- and second-tiers are both positive, it is followed by sequencing of the *ABCD1* gene for final screening [12].

In our study, we conducted a comprehensive retrospective analysis of patient records, specifically focusing on individuals who initially screened positive for X-ALD during the first two tiers, but subsequently tested negative for X-ALD through molecular analysis in the third tier. The consistently negative results in the third-tier testing across all of the analyzed cases prompted consideration for a peroxisomal disorder beyond X-ALD. Patients diagnosed with other conditions such as AGS or neonatal lupus were excluded from our analysis. This exclusion was implemented to ensure a focused investigation on peroxisomal disorders related to *PEX* genes and atypical patterns of *ABCD1* variants. Biochemical confirmatory testing was obtained within 7–30 days of age after the positive NBS was initially called out to a specialty metabolic center. All blood specimens for the measurement of VLCFA and plasmalogens were sent to the same laboratory (Kennedy Krieger Institute Laboratory in Baltimore, MD, USA). The confirmatory molecular testing included either a multigene panel that analyzed 18 genes associated with ZSD or whole exome sequencing (WES).

## 3. Results

We describe nine cases that were detected by the CA NBS program with elevated C26:0-LPC (tier one and two screening), but had negative *ABCD1* sequencing (tier three screening). Results are summarized in Table 1. For the first-tier testing, the mean C26:0-LPC result was 1.68 (cutoff ≤ 0.42 μmol/L) and the median was 2.20, with a measured range of 0.44–3.01. For the second-tier testing, the C26 mean result was 1.00 (cutoff ≤ 0.22 μmol/L) and the median was 1.18, with a measured range of 0.224–1.475.

All of the nine included cases were born full-term and only one was diagnosed with fetal growth restriction (FGR). Three of them required admission to the neonatal intensive care unit (NICU) after birth. At the time of this report submission, only six patients were alive. The three deceased patients all required NICU admission and died before one year of age, at two, four, and seven months, respectively. Of the six patients that are still alive, three are compound heterozygotes for pathogenic variants in *PEX* genes and one is homozygous for a pathogenic variant in a *PEX* gene. Two of the four *PEX*-related ZSD patients have global developmental delay (GDD) while the other two have normal development. Of the other two living non-ZSD cases, one case was diagnosed with Klinefelter syndrome (KS) and was found to have a hemizygous VUS variant in *ABCD1,* while the other non-ZSD case has a heterozygous 84 kb contiguous gene deletion on the long arm of the X chromosome including the *ABCD1* gene, which was classified as a VUS. Only one case was the product of a consanguineous couple and was homozygous for the same variant in the *PEX1* gene. It has been notoriously difficult to determine the severity based on the clinical manifestations in ZSD [13].

Regarding the encountered genetic variants, the majority have been previously reported and classified as either likely pathogenic or pathogenic in genetic databases. Notably, the *PEX1* gene was the most frequently affected in our series, with three cases exhibiting variants within this gene. Remarkably, none of the encountered variants were observed in duplicate, except in the case of consanguinity, where homozygosity was noted (case #2). Furthermore, one patient presented as a compound heterozygote for the recently identified founder variant in *PEX6* (c.1409G > C [p.Gly470Ala]), a variant associated with a severe phenotype of ZSD among individuals of Mixteco ancestry [14,15]. Additionally, our study introduced a novel pathogenic variant in the *PEX1* gene (c.2972delC; p.Pro991Leufs*9), which has not been reported in genetic databases such as ClinVar and Gnomad.

From a therapeutic standpoint, four cases (#1, #3, #5, and #8) received cholic acid treatment starting between two and eight months of age (Table 2). Case #5 discontinued treatment after a few weeks due to gastrointestinal side effects. Case #3 died at four months from multiorgan failure despite the commencement of cholic acid at two months while still under NICU care. Case #1 had an initial increase in transaminases and γ-glutamyltransferase (GTT) that decreased after 6 months of treatment. In the newborn period, Case #8 had mildly elevated transaminases, which remained elevated through 8 months of age when cholic acid was initiated; transaminases returned to normal by 14 months.

## 4. Discussion

X-linked adrenoleukodystrophy (X-ALD) is the most common peroxisomal disorder, and the implementation of X-ALD NBS screening has facilitated the early identification of patients with other genetic metabolic diseases beyond X-ALD that also cause an increase in C26:0-LPC including Zellweger spectrum disorder (ZSD). Notably, ZSD cases have demonstrated significantly elevated C26:0-LPC levels in NBS, highlighting its utility as a biomarker for peroxisomal disorders. ZSD presents a diagnostic challenge due to its heterogeneous clinical manifestations. Our study provides insights into the biochemical, molecular, and clinical characteristics of ZSD cases identified through NBS and provides a glance of its therapeutic management.

It is notable to emphasize that our study encompassed two outlier cases (Cases #6 and #7) exhibiting slightly elevated C26:0-LPC levels on both first- and second-tier testing compared to the remaining seven cases. This discrepancy stems from genetic variants that do not impact *PEX* genes, distinguishing them from the remaining cases. Upon the exclusion of these outliers, recalibration of our data revealed an increase in the average C26:0-LPC levels on the first and second tiers. Specifically, the first-tier C26:0-LPC average level rose to 2.02 (cutoff of <0.42 μmol/L), while the second-tier C26:0-LPC average level increased to 1.20 (cutoff of <0.22 μmol/L). Notably, these recalibrated averages highlight a significant elevation, with the first-tier result being 4.8 times higher and the second-tier result being 5.4 times higher than their respective cutoff values.

Of the nine patients included in our cohort, only six were alive at the time of report submission. Only two cases (#2 and #6) had intrauterine growth restriction (IUGR) and were born small for gestational age (SGA), with birth weight < 10th percentile for age and sex. From the organ-system assessment perspective, only two cases had a liver US performed and both had non-specific findings. In addition, only one case had abnormal eye findings but the three deceased cases did not have an eye evaluation given their severity and eventual death. Case #1 is currently on therapy with cholic acid, which was started at three months of age. Despite the early commencement, his liver markers such as transaminases, GTT, and direct bilirubin have remained persistently elevated, indicating ongoing liver damage. The patient has been making slow but steady developmental progress. Case #9, who harbors the Mixteco founder variant in one allele, was initiated on cholic acid but this treatment had to be interrupted due to gastrointestinal (GI) side effects. Nonetheless, the patient remains alive and thus far exhibits favorable developmental outcomes. The two non-ZSD cases did not receive cholic acid treatment. Analysis of their management revealed inconsistency in the criteria and age for commencing cholic acid therapy, limiting conclusions about its potential efficacy in this condition. The transient increase in transaminases in Case #1 had been reported in other cases on cholic acid before their improvement [16].

We emphasize the importance of X-ALD screening given that a secondary benefit has been observed by detecting individuals with other metabolic conditions beyond X-ALD. The greatest benefits of detecting ZSD through NBS include earlier detection prompting earlier organ-system investigation and therapeutic intervention; definitive diagnosis, especially for families who have had previously affected children that died without diagnosis as in two of our cases; and reproductive choices for future desired pregnancies.

## 5. Conclusions

Our study highlights the importance of detailed biochemical and molecular analyses following a positive newborn screen for XALD as screening for this primary genetic condition can identify other disorders with heterogenous genetic causes and clinical manifestations, especially ZSD. As per recent data, there are currently seven states in the USA that do not screen for XALD (https://adrenoleukodystrophy.info/clinical-diagnosis/ald-newborn-screening, accessed on 11 June 2024). As states move to expand their screening programs to encompass all those conditions on the RUSP such as X-ALD, they should be aware that there are other conditions that could be identified. Outside the U.S., other countries such as Taiwan, the Netherlands, Japan, and Italy have initiated ALD newborn screening programs [17,18].

This study was not prospectively designed to assess the effect of treatment in this population, therefore, our results are limited by the available information of the retrospective analysis. We pose the hypothesis that the earlier detection of ZSD cases will prompt the earlier initiation of treatment (i.e., cholic acid), leading to better clinical outcomes. However, a longer follow-up study is required to test the efficacy of earlier treatment intervention after the newborn screening is highly suggestive of ZSD and X-ALD has been ruled out.

As the screening for X-linked adrenoleukodystrophy continues through the measurement of C26:0-LPC, our knowledge and understanding of additional genetic conditions associated with elevated levels of this biomarker or other VLCFA will inevitably expand. This continuous advancement in knowledge will facilitate a broader recognition of other genetic disorders that present with elevated C26:0-LPC, thereby enhancing our capacity for early identification and intervention in cases where prompt medical attention is imperative. Consequently, this evolving comprehension will potentially serve to improve patient outcomes and their quality of life.

## Figures and Tables

**Table 1:** Newborn screening (NBS) results, molecular testing results, and a summary of the clinical features of the nine patients at last assessment.

Newborn Screening (NBS) Results	Case #1	Case #2	Case #3	Case #4	Case #5	Case #6	Case #7	Case #8	Case #9
NBS 1st-tier C26 μmL/L (Cutoff ≤ 0.42)	2.29	2.20	3.01	1.79	1.05	0.44	0.54	1.95	1.85
NBS 2nd-tier C26 μmL/L (Cutoff ≤ 0.22)	1.28	1.178	1.239	1.475	0.903	0.224	0.33	1.041	1.34
NBS 3rd-tier *ABCD1* sequencing	Negative	Negative	Negative	Negative	Negative	None	Heterozygous VUS in *ABCD1* (see below)	None	VUS in *ABCD1*, c.1489-6delC
**Molecular testing**									
Variant 1	*PEX1*, c.2972delC (p.Pro991Leufs*9) PV	*PEX6*, c.1233 + 3G > C VUS	*PEX1*, c.2368C > T (p.Arg790*) PV	*PEX12*, c.625C > T (pGln209*) PV	*PEX6*, 1409G > C (p.Gly470Ala) PV	**84 kb contiguous deletion of Xq28 including the *ABCD1, BCAP31, SLC6A8* and *PLXNB3* genes**	***ABCD1* (c.576 C > A (p.Asn192Lys)** **hemizygous VUS**	*PEX6* (c.2095-21_2095-10del) PV	*PEX1* (c.2391_2392del, p.Arg798Serfs*35) LPV
Variant 2	*PEX1*, c.2528G > A (p.Gly843Asp) PV	*PEX6*, c.1233 + 3G > C VUS	*PEX1*, c.3152_3156del (p.Leu1051Glnfx*24) PV	*PEX12*, c.268_271delAAGA (p.Lys90Glufs*3) LPV	*PEX6*, c.2735C > T (p.Ala912Val) PV	None	None	*PEX6* c.1313T > C (p.L438P) VUS	*PEX1* (c.2966T > C, p.Ile989Thr) PV
**Clinical Status**	**Case #1**	**Case #2**	**Case #3**	**Case #4**	**Case #5**	**Case #6**	**Case #7**	**Case #8**	**Case #9**
**Alive**	Yes	No	No	No	Yes	Yes	Yes	Yes	Yes
**IUGR**	No	Yes	No	No	No	Yes	No	No	No
**Age at last f/up**	11 m	2 m	2 m	5 m	4 m	33 m	34 m	10 m	10 m
**Current age or age at death**	12 m	4 m	2 m	7 m	5 m	37 m	38 m	14 m	68 m
**Height (percentile)**	<1st	<1st	38th	<1st	43rd	45th	89th	5th	25th
**Weight (percentile)**	<1st	<1st	<1st	<1st	83rd	60th	48th	<1st	10th
**Head circumference (percentile)**	3rd	NA	<1st	3rd	46th	51st	38th	76th	NA
**Development**	GDD	GDD	NA	GDD	Normal	Normal	Normal	Normal	Normal
**Newborn hearing screening**	Failed NB hearing screen and repeat hearing test. Wearing hearing aids	Failed NB hearing screen	NA	Failed NB hearing screen	Failed NB hearing screen and repeat hearing test	Failed NB hearing screen unilaterally. Normal hearing exam at 4 m	Failed NB hearing screen but later passed at his outpatient hearing test	Failed NB hearing screen unilaterally, which he later passed	Passed
**Renal**	US: normal	US: Mild right side hydronephrosis	US: bilateral mild hydronephrosis and echogenic kidneys	Initial US: Multiple tiny cysts throughout R kidney. On f/up US: small bilateral renal cysts	Renal US not performed	Renal US not performed	Renal US not performed	US: normal	US: normal
**Liver**	Cholestasis, elevated transaminases	Cholestasis, elevated transaminases. Liver failure	Cholestasis, elevated transaminases. Liver failure	Cholestasis, elevated transaminases	Cholestasis, elevated transaminases	ND	Normal	Elevated transaminases	Elevated transaminases
**Ophthalmologic**	Progressive opacity in lenses and cornea, pigmentary retinopathy	ND	ND	ND	No lenses, corneal or retinal lesions	ND	No lenses, corneal or retinal lesions	No lenses, corneal or retinal lesions	No lenses, corneal or retinal lesions
**Hypotonia**	Profound	Profound	Profound	Profound	Mild	No	No	No	No
**Seizures**	No	No	Yes	Yes	No	No	No	No	No
**Brain MRI**									
**Age performed**	1 m	2 wk	ND	2 wk	ND	ND	14 m. Subsequent at 20, 26, 34 m	ND	ND
**Findings**	Brachycephaly and bilateral subependymal cyst	Mild prominence of the lateral ventricles	ND	Colpocephaly, left greater than right; thinning of the corpus callosum; mild to moderate white matter volume loss	ND	ND	Chronic microhemorrhages in the cerebellar hemispheres. Nonspecific signal abnormality in the left periatrial white matter	ND	ND

Note: Bolded results represent non-ZSD cases with atypical patterns in *ABCD1*. Legend: m, months; wk; weeks; IUGR: intrauterine growth restriction; ND, not done; US, ultrasound; PV, pathogenic variant; LPV, likely pathogenic variant; VUS, variant of uncertain significance; fs* frameshift termination.

**Table 2:** Summary of cholic acid therapy.

	Case #1	Case #2	Case #3	Case #4	Case #5	Case #6	Case #7	Case #8	Case #9
**Cholic acid**	Yes	No	Yes	No	Yes	No	No	Yes	No
**Age when started**	3 m	NA	2 m	NA	5 m	NA	NA	8 m	N/A
**Dose**	100 mg/d	NA	100 mg/d	NA	100 mg/d	NA	NA	100 mg/d	N/A
**G-tube fed**	No	No	No	Yes	No	No	No	No	No

Legend: m, months; NA, not applicable.

## Data Availability

The raw data supporting the conclusions of this article will be made available by the authors on request.
